# Revisiting the Effect of Capture Heterogeneity on Survival Estimates in Capture-Mark-Recapture Studies: Does It Matter?

**DOI:** 10.1371/journal.pone.0062636

**Published:** 2013-04-30

**Authors:** Fitsum Abadi, Andre Botha, Res Altwegg

**Affiliations:** 1 South African National Biodiversity Institute, Claremont, South Africa; 2 Animal Demography Unit, Department of Biological Sciences and Department of Statistical Sciences, University of Cape Town, Rondebosch, South Africa; 3 Endangered Wildlife Trust, Modderfontein, South Africa; Australian Wildlife Conservancy, Australia

## Abstract

Recently developed capture-mark-recapture methods allow us to account for capture heterogeneity among individuals in the form of discrete mixtures and continuous individual random effects. In this article, we used simulations and two case studies to evaluate the effectiveness of continuously distributed individual random effects at removing potential bias due to capture heterogeneity, and to evaluate in what situation the added complexity of these models is justified. Simulations and case studies showed that ignoring individual capture heterogeneity generally led to a small negative bias in survival estimates and that individual random effects effectively removed this bias. As expected, accounting for capture heterogeneity also led to slightly less precise survival estimates. Our case studies also showed that accounting for capture heterogeneity increased in importance towards the end of study. Though ignoring capture heterogeneity led to a small bias in survival estimates, such bias may greatly impact management decisions. We advocate reducing potential heterogeneity at the sampling design stage. Where this is insufficient, we recommend modelling individual capture heterogeneity in situations such as when a large proportion of the individuals has a low detection probability (e.g. in the presence of floaters) and situations where the most recent survival estimates are of great interest (e.g. in applied conservation).

## Introduction

Survival of animals in the wild is an important fitness component, and unbiased survival estimates are critical for understanding, among other things, the patterns of life histories [1], evolutionary pressures in the wild (e.g. [2]), and for the conservation of populations [3]. Development of sophisticated open capture-mark-recapture models [4] has revolutionized our knowledge of survival in populations of wild animals (reviewed by [5]). In theory, these methods give unbiased survival estimates by incorporating an estimate of the detection probability (i.e. the probability of recapture (or resighting) an individual that is alive and in the population at the time of a survey) into the estimation of survival probability. The detection probability is often regarded as a nuisance parameter, and is usually of little biological interest (but see [6]). However, as methods are now used extensively (the key publication, [4], has been cited 2109 times, according to ISI Web of Science, accessed on 14 March 2013), the importance of accounting for the detection probability is becoming clear: almost all studies find detection probabilities <1 (implying that perfect detection is hardly ever achieved). Further, most studies find the detection rate to vary among groups of individuals (e.g. age classes and sex), and over time and space. This suggests modelling variation in detection probabilities is critical for obtaining unbiased survival estimates from capture-mark-recapture experiments on wild populations [4], [7], [8].

Conventional capture-mark-recapture methods make the critical assumption of equal detection probability among individuals within a group. This assumption is generally tested using standard tests (Test 2 in RELEASE, ([9]; also available within program MARK: [10]), and U-CARE, ([11], [12])), and often found to be violated. However, since [13] found that the departure from the assumption of homogeneity in detection causes negligible bias in survival estimates, the resulting lack of fit (to which unmodelled individual heterogeneity contributes) is normally dealt with by multiplying the variance-covariance matrix by a constant variance inflation (overdispersion) factor, ĉ [4], [10]. This inflates the confidence intervals, but does not attempt to correct any potential bias in the mean estimate.

More recently, however, concerns about the assumption that capture heterogeneity in the estimation of survival and/or population size can safely be ignored have been voiced [14]–[19]. At the same time, the development in hierarchical models for analysing capture-mark-recapture data has made it possible to address this issue as capture heterogeneity can now be modelled in various ways [15], [20]–[22]. While appealing, these methods add complexity to the analyses, and are more difficult to fit and assess for general users of capture-mark-recapture methodology.

Therefore, the objectives of this study are to reassess effects of different forms of capture heterogeneity on survival estimates, and to identify situations in which the assumption of homogenous detection probability can safely be applied to empirical data sets. We use simulations and two case studies where we expected capture heterogeneity to be strong, to illustrate pros and cons of modelling capture heterogeneity in capture-mark-recapture studies.

## Methods

The family of Cormack-Jolly-Seber (CJS) models is widely used to estimate survival probabilities from capture-recapture data. This model can be implemented either using the multinomial likelihood [4], [9] or the state-space formulation [20], [23], [24]. Here, we used the latter approach as it provides a flexible framework for incorporating individual heterogeneity and easily fitting other modified models [20], [23], [25]–[28]. Before we present the model, we define the notations. Let 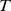 be the number of sampling occasions in year, 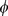 be the survival probability, which is assumed constant over time and identical for all individuals, and 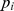 be the detection probability of individual *i* and constant over time. The model is expressed by the state process, which describes the true biological process (e.g. survival), and the observation process, which describes the error associated with the data (e.g. imperfect detection). Following [20], [23], the state process (i.e. 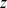 = 1 (alive) or 0 (dead) for individual *i* at time *t*) is described by Bernoulli trials as
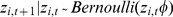
(1)


The state of an individual at the time of first capture is known with probability 1 (i.e. it is alive with certainty). The observation process (i.e. *y* = 1 (seen alive), 0 (not seen) for individual *i* at time *t*) is modelled by Bernoulli trials as
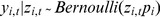
(2)


To assess the effect of ignoring capture heterogeneity, we considered a model that allowed for capture heterogeneity ({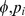}) vs. a model that assumed constant detection probability across individuals ({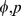}). In the former, we used a random effects model to account for capture heterogeneity, and individual random terms (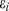) are the deviations from the global mean 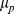, which are distributed normally with a mean of 0 and standard deviation 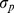. That is,
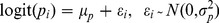
(3)


### 1. Simulation Study

**Figure 1:**
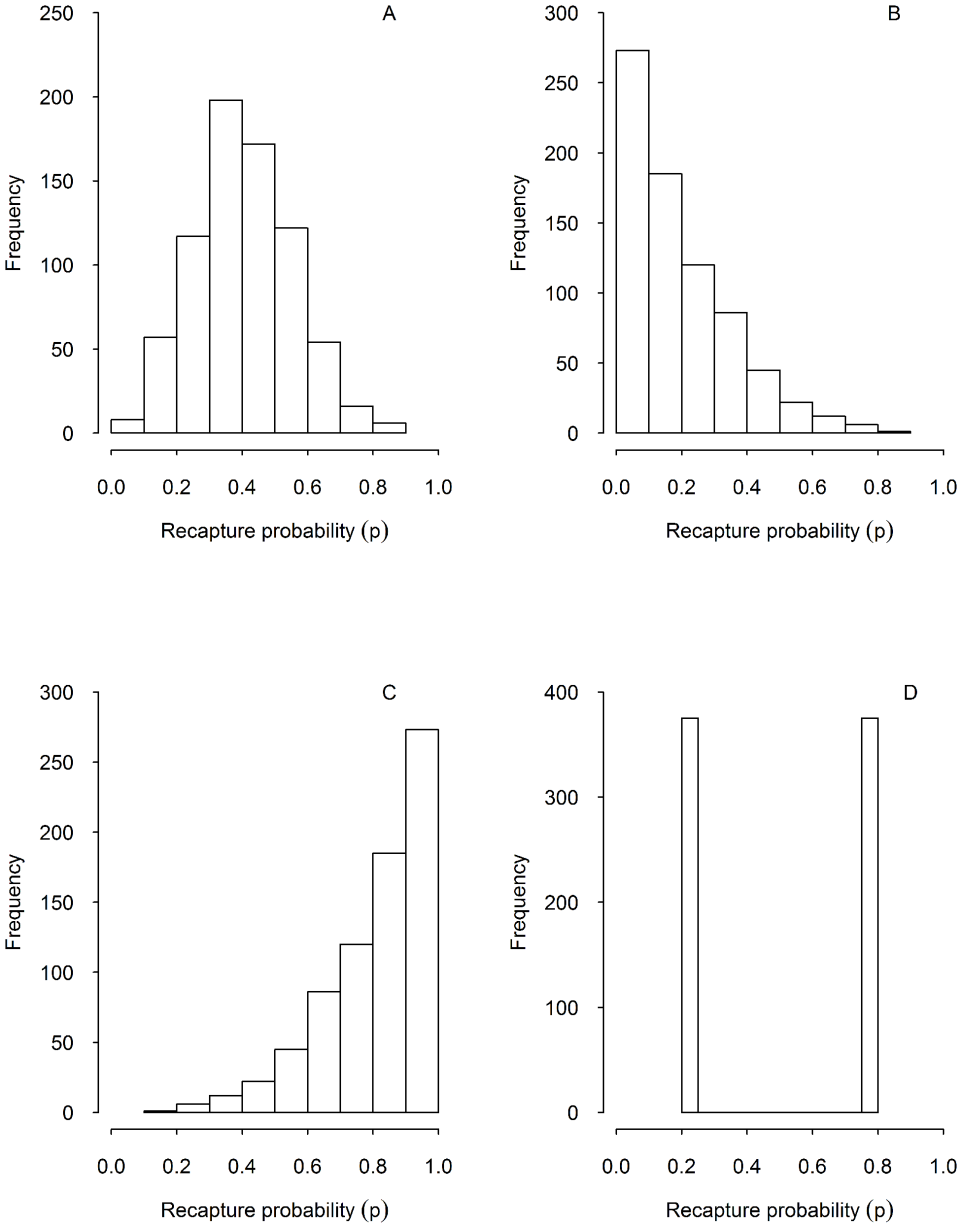
Different scenarios of heterogeneity in detection probabilities. a) Symmetric individual detection probabilities, b) right-skewed (most individuals had a lower detection probability), c) left-skewed (most individuals had a higher detection probability), and d) two-group heterogeneity (individuals with low and high detection probabilities).

**Figure 2:**
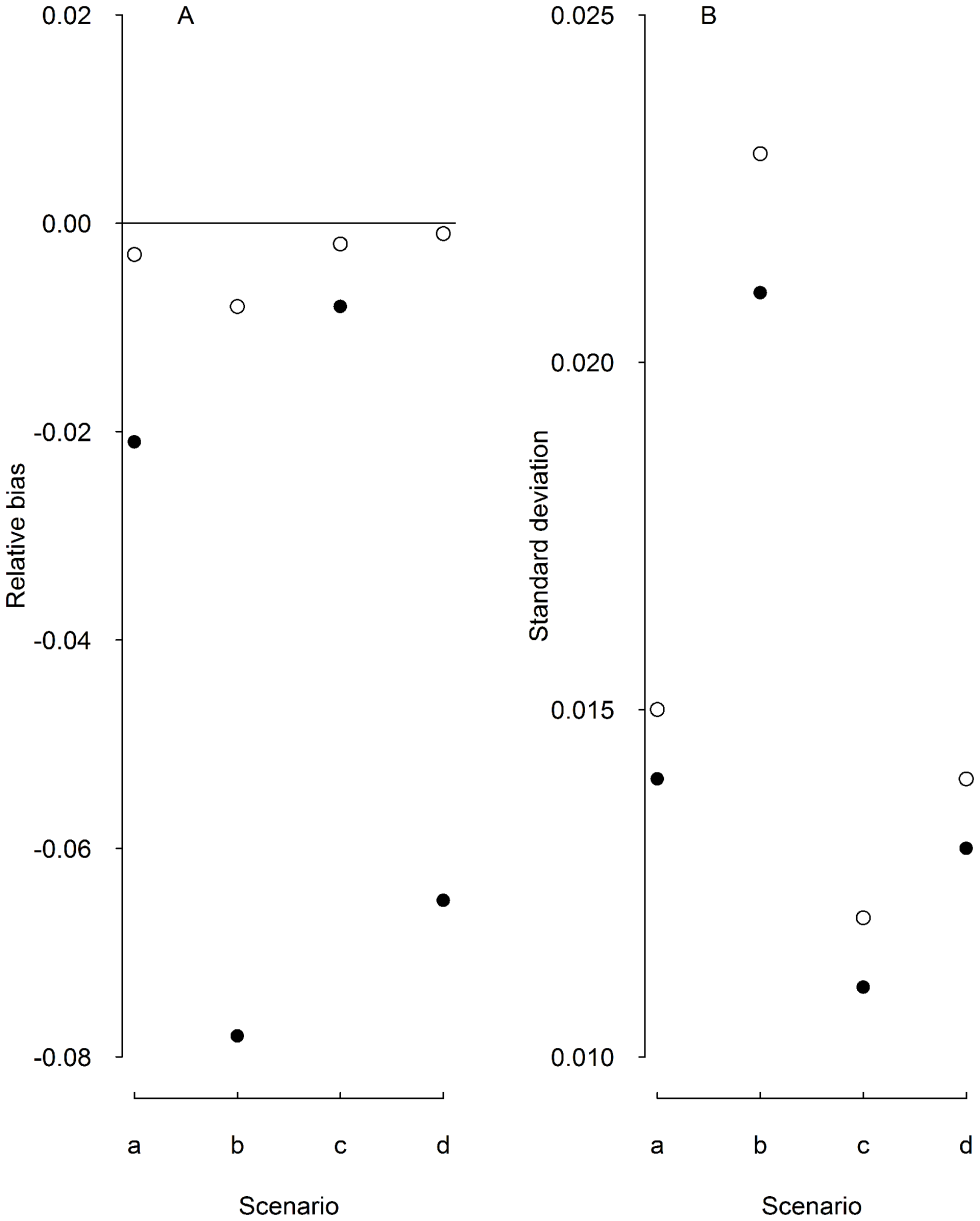
Relative bias and precision in the estimate of survival probability. The relative bias (panel 1) in the estimate of survival probability and precision in terms of standard deviation (panel 2) from a model that ignores (solid circle) and accounts for heterogeneity (open circle) in detection probability under different scenarios: a) symmetric individual heterogeneity, b) right skewed, c) left skewed, and d) two-group heterogeneity.

**Figure 3:**
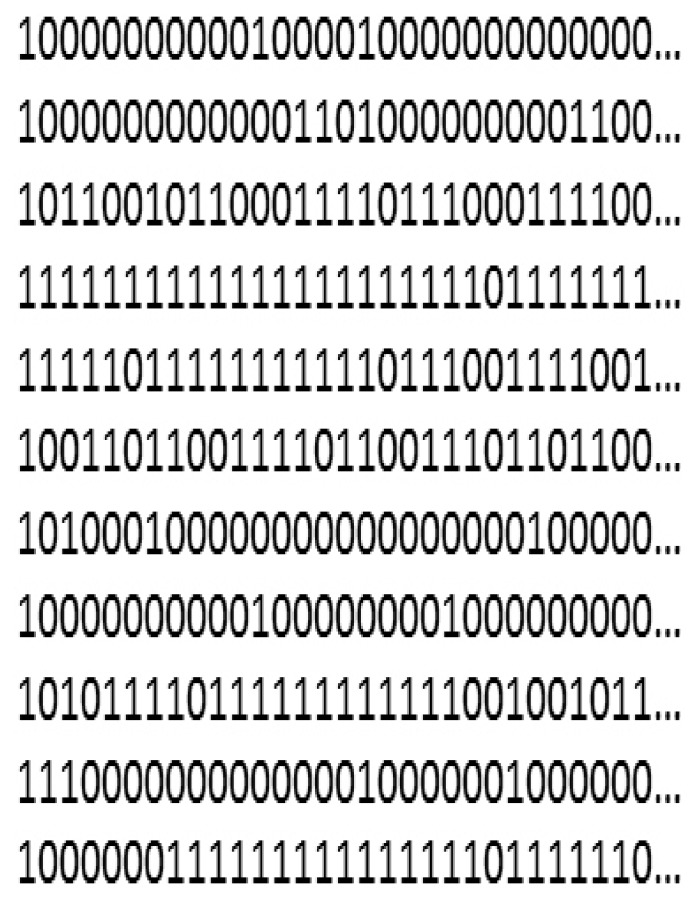
Example of parts of the capture-resighting histories for a subset of the African White-backed Vultures. The rows correspond to individuals and the column to months. If a particular bird was seen in a given months, its sighting history contains a ‘1′ in the corresponding column, and ‘0′ otherwise. The capture histories suggest strong heterogeneity in resighting probabilities, probably due to individual differences in movement patterns.

We carried out a simulation study to assess the potential bias in estimates of survival probability when capture heterogeneity is ignored. In conducting simulations, we first needed to specify the parameters. The number of sampling occasions in year (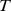) set at 15 years, the survival probability (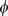) was set at 0.7 and assumed to be constant over time. We considered four different scenarios of capture heterogeneity (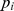), which could frequently arise in empirical capture-recapture studies: 1) symmetric heterogeneity around a mean detection probability (Figure 1a); 2) right-skewed distribution of detection probabilities (e.g. most individuals have relatively low detection probabilities but a few individuals are being caught repeatedly; a situation that could arise if the study area is relatively small in relation to the movement patterns of the individuals) (Figure 1b); 3) left-skewed distribution (e.g. most individuals have relatively high detection probabilities but a few individuals are unlikely to be detected because they have their home ranges along the periphery of the study area) (Figure 1c); and 4) two-group heterogeneity, a situation if the studied population consists of two groups (e.g. females and males, non-breeders and breeders, social status) that cannot be distinguished in the field but differ in their propensity to be trapped (Figure 1d). The first three scenarios of detection probabilities were generated from a beta distribution with means 0.4, 0.2, and 0.8 and standard deviations 0.148, 0.163, and 0.163, respectively. In the fourth scenario, we considered a detection probability of 0.2 for one group and 0.8 for the other group. For each scenario, the annual number of newly marked individuals was chosen to be 50. We then simulated 100 replicate data sets each under the assumption that survival probability is constant over time and identical for all individuals, and under the assumption that detection probability varies only across individuals.

To each simulated data set, we fitted a model that allows for heterogeneity in detection probability among individuals ({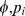}), and a model that assumes constant detection probability across individuals ({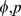}). The former model is equivalent to the generating model for the first scenario, with symmetric heterogeneity. We used this model ({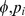}) to analyse all data sets, including the ones generated under the other scenarios for heterogeneity, to see how well this approach works in different situations.

All the analyses were performed within the Bayesian framework, specifying non-informative priors to reflect little *a priori* knowledge about the parameters. We used uniform prior distributions between 0 and 1 (U(0,1)) for the survival and mean detection probabilities, and a U(0,10) prior distribution for the standard deviation of the detection probabilities. We first assessed the convergence of the Markov Chain Monte Carlo (MCMC) algorithm to the targeted posterior distribution by running 3 chains of 10000 iterations with a burn-in of 7000. The 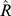 [29] values were below 1.01 for all parameters, indicating convergence. We then ran a single chain of 30000 iterations, a burn-in of 20000, and retained every 10^th^ observation for each simulated data. Thus, the posterior summary statistics were computed based on 1000 MCMC samples. We then calculated the relative bias in estimates of survival probability as
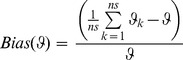
(4)where 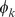 is the estimated survival probability for the 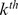 simulated data, 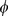 is the survival probability used to generate the data, and 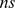
is the number of simulated data sets. Further, we assessed the effect of ignoring detection heterogeneity on the precision of survival estimates. The data simulation was carried out using R [30] and the analysis was performed using WinBUGS calling from R using R2WinBUGS [31].

#### Simulation results: Bias and precision of survival estimates

Ignoring heterogeneity in detection probability led to negative bias in estimates of survival probability (Figure 2a). Though the bias was small in all scenarios, our analyses revealed the largest bias in the cases of the right-skewed scenario (i.e., most individuals had relatively low detection probabilities) and two groups with different detection probabilities. A model accounting for heterogeneity essentially produced unbiased estimates of survival probability in all cases (Figure 2a). As expected, the model that allows for heterogeneity in detection probability provided slightly less precise (i.e. large standard deviation) estimates of survival probability compared to the one ignoring it (Figure 2b). The individual random effects model slightly underestimated the standard deviations of the detection probabilities (with relative bias ranging from −0.19 to −0.05).

#### Goodness-of-fit (GOF) tests

To see whether commonly used diagnostics would flag the detection heterogeneity in our simulations, we randomly chose five simulated data sets per scenario and tested for detection heterogeneity in program U-CARE [11], [12]. The overall GOF test (i.e., TEST3.SR+TEST3.SM+TEST2.CT+TEST2.CL) for the CJS model showed little evidence for lack of fit at the 5% significance level except for the scenario with two groups differing in detection probabilities (Appendix S1). For this scenario, the directional tests for both transience and trap dependence were significant (all p-values<0.05). For all selected data sets, the estimated overdispersion parameter (ĉ) varied between 1.000 and 2.162, suggesting evidence of overdispersion (Appendix S1). Interestingly, for the second scenario (Figure 1b), the TEST2.CT test showed signs of transience whereas for the third scenario (Figure 1c), the TEST3.SR test for trap dependence was statistically significant (Appendix S1).

### 2. Case Studies

Our simulation study clearly showed that ignoring capture heterogeneity led to a small bias in survival estimates and a slight overestimation of its precision (i.e. small standard deviation). We also examined the issue of capture heterogeneity in two case studies involving data on African White-backed Vultures (*Gyps africanus*) and on African Penguins (*Spheniscus demersus*). While we advocate reducing capture heterogeneity by choosing a sampling design that minimizes the problem, this is not always possible and our case studies are examples of the latter. Both species are of conservation concern and subject to tagging programs that can be used to assess effective management strategies. In both data sets, we expected strong detection heterogeneity for reasons detailed below, and we assessed the extent to which this may lead to biased survival.

#### Ethics statement

Capturing and tagging of the birds was done under permits from SAFRING, CapeNature, and the Department of Environmental Affairs (DEA). Under South African laws, when in possession of a research permit allowing banding of penguins, no additional ethics clearance is required for these birds. They were captured by hand and stainless steel flipper bands were applied according to the guidelines approved by the DEA [32], who was also responsible for ethical oversight. Stainless steel bands are tear-drop shaped and the ends overlap, allowing each band to be individually fitted to the penguin using custom-made banding pliers. The penguins were banded by officials of the DEA and the South African Bird Ringing Unit (SAFRING) with permits issued under the Sea Birds and Seals Protection Act No. 46 of 1973, the Marine Living Resources Act No. 18 of 1998, and the National Environmental Management Biodiversity Act No. 10 of 2004. Both institutions agreed on the use and publication of these data. The vultures were fitted with patagial tags, for which ethics clearance was provided by the Endangered Wildlife Trust Ethics Committee and the ethics committee at South African National Parks. The effect of tagging was minimised by adopting the standard protocol adopted for this practice in southern Africa [33].

#### The African White-backed Vulture Study

Ninety-three vultures were captured using carcass baited walk in traps between November 2005 and January 2007 at the Moholoholo Wildlife Centre near Kampersrus in Mpumalanga, South Africa. Captured birds were fitted with unique alphanumerically coded patagial tags and standard metal leg rings. Individuals were then resighted monthly between December 2005 and June 2010 near the capture site where vultures were being fed and also within the Greater Kruger National Park where the birds are known to forage. Re-sightings away from the capture site were reported by members of the public visiting or staff working within the Kruger National Park and adjacent nature reserves. While some vultures visited this site regularly, others were only found there occasionally, thus creating strong individual heterogeneity in resighting probabilities (Figure 3). See [34] for more details.

#### The African Penguin study

We analysed a capture-mark-recapture data set consisting of 5558 adult African Penguins banded and resighted on Robben Island, South Africa, between 2002 and 2009. Birds returning to their breeding colony were observed with a spotting scope and their flipper bands read. The birds use different paths to walk from the beach to the colony and even though flipper bands were read in all areas that penguins used, most of the resighting effort was concentrated on two main paths used by most penguins. We expected the spatial heterogeneity in effort to translate into individual resighting heterogeneity if penguins consistently used the same areas in their colony, as is the norm for seabirds. There is also the possibility of unidentified groups with different resighting probabilities as we could not tell apart the sexes in the field and a range of band types of different quality were used [35], even though the latter effect could have been incorporated into the model structure. More information on the penguin study can be found in [36].

For both data sets, we fitted a model allowing for individual heterogeneity in resighting probabilities and one assuming constant resighting probabilities, using the same methods as for analysing the simulated data above. The models further allowed for a time effect on survival and resighting probabilities, treating time as a random effect in the longer vulture study and as a fixed effect in the shorter penguin study. We computed the posterior summary statistics using a single chain of 200000 iterations after discarding the initial 100000 iterations as a burn-in period and thinned by using every 10^th^ observation. The R and WinBUGS codes used for fitting all models are available on request.

#### Case studies results

**Figure 4:**
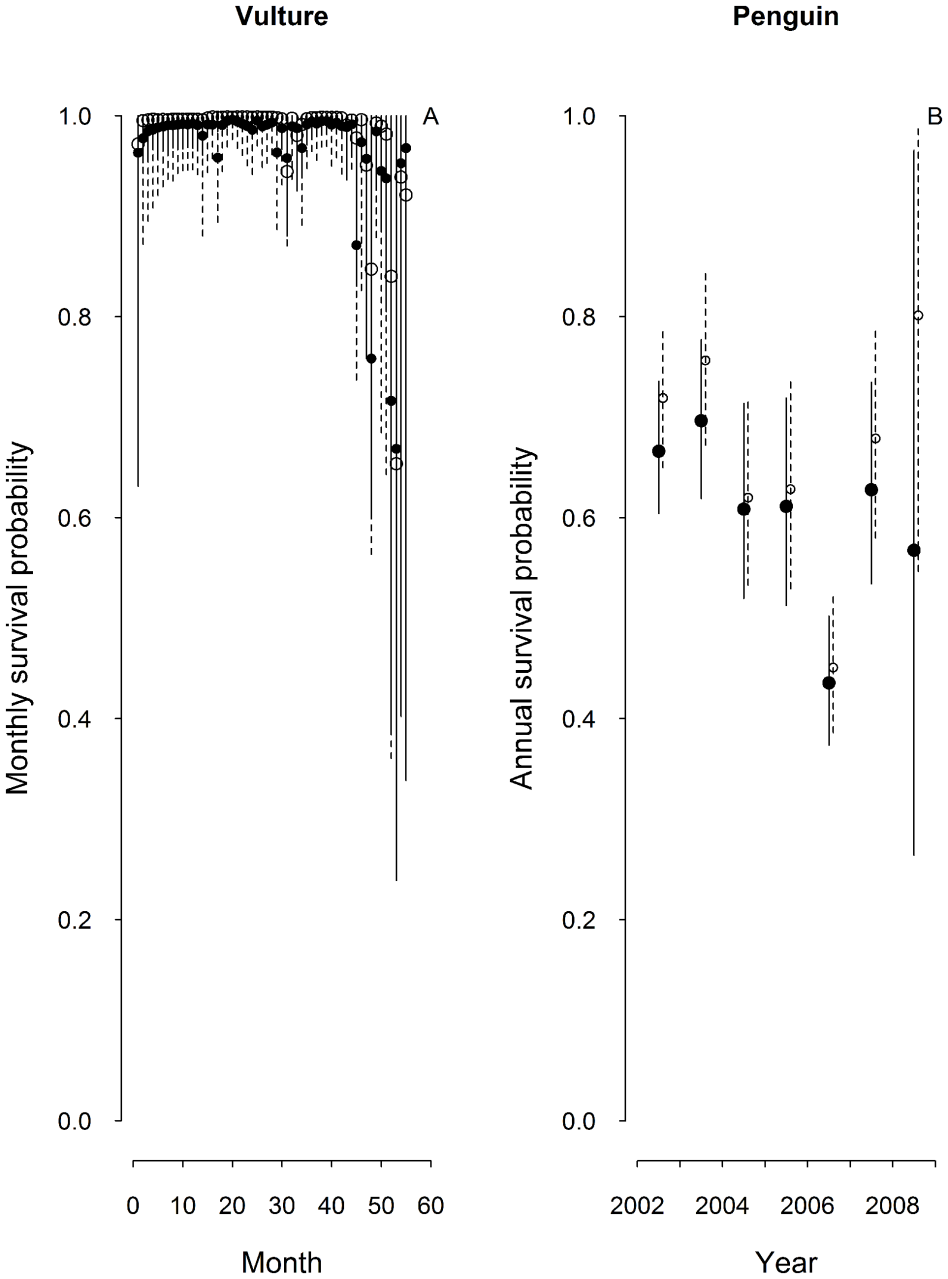
Estimates of survival probability. Mean survival estimates along with the 95% credible interval obtained from a model that ignores heterogeneity (solid symbols and lines) and a model that allows for heterogeneity (open symbols and broken lines) for (a) the African White-backed Vultures data, and (b) the African Penguins data.

For the African White-backed Vulture data, the estimated monthly survival probabilities were close to 1 except for a few months (Figure 4a). The posterior distribution of the standard deviation of monthly individual resighting probability (on the logit scale) had a mean of 1.78 (95% CRI: 1.50–2.10), indicating evidence of heterogeneity in resighting probabilities. However, both models, the one ignoring heterogeneity and the one allowing for it, provided similar estimates of survival probabilities for most months of the study (Figure 4a). The overall GOF test from U-CARE indicated a serious lack of fit (χ^2^ = 627.903, df = 142, P<0.001) with the largest contribution coming from TEST2.CT (χ^2^ = 487.068, df = 53, P<0.001). The directional test for transience was statistically significant and the ĉ obtained from the GOF test showed substantial overdispersion (ĉ = 4.422). In general, the model ignoring heterogeneity yielded survival estimates with shorter confidence intervals than the one accounting for it (Figure 4a). For African Penguins, the mean annual survival probabilities varied between 0.530 and 0.817, and the model ignoring heterogeneity tended to underestimate the survival probability in some years (Figure 4b). Our analysis revealed that the mean of the posterior distribution of the standard deviation of the resighting probability was 1.239 (95% CRI: 0.883–1.637), suggesting evidence of heterogeneity in resighting individuals. The overall GOF test result from U-CARE showed overdispersion (χ^2^ = 83.924, df = 28, P<0.001), and TEST2.CT was highly significant (χ^2^ = 37.292, df = 5, P<0.001). The directional test for transience was statistically significant and the ĉ obtained from the GOF test showed overdispersion (ĉ = 2.997). Yet, ignoring this variation only had a small effect on the survival estimates and the precision of the survival estimates was comparable for both models (Figure 4b).

## Discussion

The traditional capture-mark-recapture modelling framework assumes constant survival and detection probabilities across individuals [4], [37]–[39]. This is of course never strictly true in real situations, but early studies showed that heterogeneity among individuals results in only slight bias in survival estimates [13]. More recently, doubts have been raised whether individual heterogeneity can safely be ignored [14], [19], [40] and methods to account for such heterogeneity have been developed [15], [21]. Individual heterogeneity can conveniently be modelled as individual random effects when formulating the model as a state-space process [20], [23] but popular software packages also offer individual random effects within the classical capture-mark-recapture modelling framework (recent versions of MARK [10] and E-SURGE [21]). We used simulations and two case studies to examine in what situations the added complexity of individual random effects is necessary, and what the costs and gains may be.

In our simulations even large individual heterogeneity in detection probabilities caused little bias in survival estimates. The largest negative biases occurred in the cases of right-skewed heterogeneity (i.e., most individuals had relatively low detection probabilities) and with two groups that differ in detection probabilities. Intuitively, the negative bias arises because the detection estimate is dominated by individuals that are detected more frequently because their detection probability is high. This leads to positive bias in detection estimates and negative bias in the survival estimates. If it was known or suspected that detection heterogeneity was due to a small number of unrecognized groups that differ in detection probabilities, this structure could be modelled using discrete mixtures [40]. In all cases considered, however, modelling heterogeneity as continuous individual random effects essentially eliminated bias. The results of this simulation are thus in agreement with [13] who found small negative biases of unmodelled capture heterogeneity on survival estimates. [16] also found small negative biases in a situation with unmodelled two-group heterogeneity. However, such small negative bias in survival estimates may still have a strong impact on the ability to select the correct management measures, in particular for long-lived species where population dynamics is very sensitive to survival [19].

Our case studies supported the results observed in our simulations. Survival estimates slightly increased when we included individual random effects on detection with greater influence towards the end of the time series. This may be due to individuals with low detection probability ‘disappearing’ towards the end of the study, which would make the estimated detection probability increasingly influenced by the individuals that are easy to detect, and thus lead to survival estimates that are increasingly biased low towards the end of the study [16]. In applied conservation the most recent survival estimates are often the most interesting ones, because they are needed to gauge the effectiveness of conservation interventions or to predict future population declines. We recommend individual random effects to be explored in such situations, particularly if detection probabilities are low. Other strategies for reducing detection heterogeneity (see below) should also reduce the problem of apparently declining survival estimates towards the end of the study.

Precision of survival estimates is also of concern when capture heterogeneity is unmodelled. Such heterogeneity could lead to confidence intervals that are too narrow due to failure to account for uncertainty in detection probabilities. In our simulations, we found that the survival estimates became slightly less precise when we used individual random effects to account for detection heterogeneity, demonstrating that ignoring detection heterogeneity can lead to optimistic confidence intervals. In the vulture case study, the model without individual random effects led to increased precision of survival estimates with confidence intervals that appear to be overly optimistic. However, in the penguin case study, adding individual random effects had little influence on precision of survival estimates.

We advocate a three-stage strategy to dealing with potential capture heterogeneity. The first stage is to reduce detection heterogeneity by study design, e.g. by standardising field effort across time and space. However, even constant effort is not likely to yield constant detection probabilities as factors beyond the control of the observer can still vary. Furthermore, constant effort may not be possible or practical. As a second stage, we therefore recommend incorporating suspected sources of heterogeneity into the model, e.g. by using covariates that are thought to be related to detection probability. Indirect information on possible hidden heterogeneity (e.g. sex determined by uncertain cues [41]) can be used in multi-event models [42]. As a third stage, we recommend modelling individual heterogeneity where there is evidence for such heterogeneity to remain, for example from a high estimate of ĉ or significant goodness-of-fit results. Our simulations showed that heterogeneity can lead to apparent trap effects and transience. Using continuously distributed individual random effects may yield better survival estimates, in terms of bias and precision than applying an overdispersion factor.

In summary, our results suggest that individual detection heterogeneity only has small effects on survival estimates. In practice, the situation that is most likely to bias survival is if a considerable proportion of the individuals have a low detection probability, like our right skewed and two-group scenarios. Transients or the presence of floaters would have such an effect. The effect of transients is well recognised and accounted for by assuming that these individuals are never recaptured [43], [44]. Even though we did not explicitly examine a scenario where some individuals have zero recapture or resighting probability, our results suggest that continuously distributed individual random effects effectively eliminate the bias due to various types of individual heterogeneity. Our results also suggest that individual random effects can improve survival estimates towards the end of the study if detection heterogeneity is present.

## Supporting Information

Appendix S1 **Summary of the GOF test results from Program U-CARE** [**12**] **.**
(DOCX)
