# Supplementary material for: Revisiting the Effect of Capture Heterogeneity on Survival Estimates in Capture-Mark-Recapture Studies: Does It Matter?
Source: PLoS One. 2013 Apr 30;8(4):e62636. doi: 10.1371/journal.pone.0062636 (PMC3639964; doi:10.1371/journal.pone.0062636)
Supplement: Appendix S1 — Summary of the GOF test results from Program U-CARE [12] . (DOCX) [file pone.0062636.s001.docx]

Appendix S1: Summary of the GOF test results from Program U-CARE (Choquet et al. 2009)

|  | **Simulation study** | | | | | | | |
| --- | --- | --- | --- | --- | --- | --- | --- | --- |
|  | **GOF tests using U-CARE** | | | | | | | |
| Scenario | data set | Tests | c-hat | df | quad chi2 | p-value | P-value for transience | P-value for trap dependence |
| **(see Fig. 1)** |  |  |  |  |  |  |  |  |
| **a) Symmetry** | c1_1 | TEST3.SR |  | 13 | 15.409 | 0.283 | 0.002 |  |
|  |  | TEST2.CT |  | 12 | 14.047 | 0.298 |  | 0.134 |
|  |  | TEST3.SM |  | 22 | 25.168 | 0.289 |  |  |
|  |  | TEST2.CL |  | 17 | 10.190 | 0.895 |  |  |
|  |  | Sum of TESTs | 1.013 | 64 | 64.815 | 0.448 |  |  |
|  |  |  |  |  |  |  |  |  |
|  | c1_2 | TEST3.SR |  | 13 | 16.360 | 0.230 | 0.027 |  |
|  |  | TEST2.CT |  | 12 | 19.718 | 0.073 |  | 0.610 |
|  |  | TEST3.SM |  | 18 | 10.789 | 0.903 |  |  |
|  |  | TEST2.CL |  | 11 | 7.217 | 0.781 |  |  |
|  |  | Sum of TESTs | 1.002 | 54 | 54.083 | 0.471 |  |  |
|  |  |  |  |  |  |  |  |  |
|  | c1_3 | TEST3.SR |  | 13 | 10.206 | 0.677 | 0.149 |  |
|  |  | TEST2.CT |  | 12 | 10.268 | 0.593 |  | 0.697 |
|  |  | TEST3.SM |  | 20 | 36.075 | 0.015 |  |  |
|  |  | TEST2.CL |  | 12 | 10.211 | 0.597 |  |  |
|  |  | Sum of TESTs | 1.171 | 57 | 66.759 | 0.177 |  |  |
|  |  |  |  |  |  |  |  |  |
|  | c1_4 | TEST3.SR |  | 13 | 5.246 | 0.969 | 0.220 |  |
|  |  | TEST2.CT |  | 12 | 15.274 | 0.227 |  | 0.165 |
|  |  | TEST3.SM |  | 18 | 30.631 | 0.032 |  |  |
|  |  | TEST2.CL |  | 13 | 20.701 | 0.079 |  |  |
|  |  | Sum of TESTs | 1.283 | 56 | 71.852 | 0.075 |  |  |
|  |  |  |  |  |  |  |  |  |
|  | c1_5 | TEST3.SR |  | 13 | 13.251 | 0.429 | 0.402 |  |
|  |  | TEST2.CT |  | 12 | 10.559 | 0.567 |  | 0.072 |
|  |  | TEST3.SM |  | 18 | 24.400 | 0.142 |  |  |
|  |  | TEST2.CL |  | 12 | 15.664 | 0.207 |  |  |
|  |  | Sum of TESTs | 1.161 | 55 | 63.874 | 0.193 |  |  |
| **b) Right-skewed** | c2_1 | TEST3.SR |  | 13 | 40.614 | 0.000 | 0.000 |  |
|  |  | TEST2.CT |  | 12 | 11.642 | 0.475 |  | 0.654 |
|  |  | TEST3.SM |  | 16 | 20.308 | 0.207 |  |  |
|  |  | TEST2.CL |  | 12 | 8.864 | 0.715 |  |  |
|  |  | Sum of TESTs | 1.536 | 53 | 81.428 | 0.007 |  |  |
|  |  |  |  |  |  |  |  |  |
|  | c2_2 | TEST3.SR |  | 13 | 15.139 | 0.299 | 0.012 |  |
|  |  | TEST2.CT |  | 12 | 10.996 | 0.529 |  | 0.188 |
|  |  | TEST3.SM |  | 12 | 10.296 | 0.590 |  |  |
|  |  | TEST2.CL |  | 13 | 19.839 | 0.099 |  |  |
|  |  | Sum of TESTs | 1.125 | 50 | 56.270 | 0.252 |  |  |
|  |  |  |  |  |  |  |  |  |
|  | c2_3 | TEST3.SR |  | 13 | 31.843 | 0.003 | 0.000 |  |
|  |  | TEST2.CT |  | 12 | 12.363 | 0.417 |  | 0.678 |
|  |  | TEST3.SM |  | 12 | 14.450 | 0.273 |  |  |
|  |  | TEST2.CL |  | 11 | 16.471 | 0.125 |  |  |
|  |  | Sum of TESTs | 1.565 | 48 | 75.126 | 0.007 |  |  |
|  |  |  |  |  |  |  |  |  |
|  | c2_4 | TEST3.SR |  | 13 | 26.056 | 0.017 | 0.003 |  |
|  |  | TEST2.CT |  | 12 | 13.414 | 0.340 |  | 0.101 |
|  |  | TEST3.SM |  | 12 | 13.556 | 0.330 |  |  |
|  |  | TEST2.CL |  | 13 | 18.379 | 0.144 |  |  |
|  |  | Sum of TESTs | 1.428 | 50 | 71.405 | 0.025 |  |  |
|  |  |  |  |  |  |  |  |  |
|  | c2_5 | TEST3.SR |  | 13 | 18.708 | 0.132 | 0.000 |  |
|  |  | TEST2.CT |  | 12 | 12.564 | 0.402 |  | 0.213 |
|  |  | TEST3.SM |  | 14 | 18.895 | 0.169 |  |  |
|  |  | TEST2.CL |  | 13 | 7.602 | 0.869 |  |  |
|  |  | Sum of TESTs | 1.111 | 52 | 57.769 | 0.271 |  |  |
|  |  |  |  |  |  |  |  |  |
| **c) Left-skewed** | c3_1 | TEST3.SR |  | 13 | 10.315 | 0.668 | 0.042 |  |
|  |  | TEST2.CT |  | 12 | 27.866 | 0.006 |  | 0.001 |
|  |  | TEST3.SM |  | 12 | 19.886 | 0.069 |  |  |
|  |  | TEST2.CL |  | 8 | 7.972 | 0.436 |  |  |
|  |  | Sum of TESTs | 1.468 | 45 | 66.039 | 0.022 |  |  |
|  |  |  |  |  |  |  |  |  |
|  | c3_2 | TEST3.SR |  | 13 | 14.428 | 0.344 | 0.284 |  |
|  |  | TEST2.CT |  | 12 | 22.602 | 0.031 |  | 0.004 |
|  |  | TEST3.SM |  | 12 | 8.465 | 0.748 |  |  |
|  |  | TEST2.CL |  | 10 | 7.328 | 0.694 |  |  |
|  |  | Sum of TESTs | 1.124 | 47 | 52.823 | 0.259 |  |  |
|  |  |  |  |  |  |  |  |  |
|  | c3_3 | TEST3.SR |  | 13 | 9.368 | 0.745 | 0.287 |  |
|  |  | TEST2.CT |  | 12 | 25.844 | 0.011 |  | <0.001 |
|  |  | TEST3.SM |  | 12 | 14.580 | 0.265 |  |  |
|  |  | TEST2.CL |  | 8 | 3.390 | 0.908 |  |  |
|  |  | Sum of TESTs | 1.182 | 45 | 53.182 | 0.188 |  |  |
|  |  |  |  |  |  |  |  |  |
|  | c3_4 | TEST3.SR |  | 13 | 8.054 | 0.840 | 0.385 |  |
|  |  | TEST2.CT |  | 12 | 18.030 | 0.115 |  | 0.002 |
|  |  | TEST3.SM |  | 12 | 7.696 | 0.808 |  |  |
|  |  | TEST2.CL |  | 11 | 16.608 | 0.120 |  |  |
|  |  | Sum of TESTs | 1.050 | 48 | 50.388 | 0.379 |  |  |
|  |  |  |  |  |  |  |  |  |
|  | c3_5 | TEST3.SR |  | 13 | 5.286 | 0.968 | 0.433 |  |
|  |  | TEST2.CT |  | 12 | 22.896 | 0.029 |  | 0.008 |
|  |  | TEST3.SM |  | 12 | 21.010 | 0.050 |  |  |
|  |  | TEST2.CL |  | 11 | 6.392 | 0.846 |  |  |
|  |  | Sum of TESTs | 1.158 | 48 | 55.584 | 0.211 |  |  |
|  |  |  |  |  |  |  |  |  |
| **d) two-group** | c4_1 | TEST3.SR |  | 13 | 33.127 | 0.002 | <0.001 |  |
|  |  | TEST2.CT |  | 12 | 22.102 | 0.036 |  | <0.001 |
|  |  | TEST3.SM |  | 16 | 23.383 | 0.104 |  |  |
|  |  | TEST2.CL |  | 10 | 17.048 | 0.073 |  |  |
|  |  | Sum of TESTs | 1.876 | 51 | 95.661 | <0.001 |  |  |
|  |  |  |  |  |  |  |  |  |
|  | c4_2 | TEST3.SR |  | 13 | 21.106 | 0.071 | <0.001 |  |
|  |  | TEST2.CT |  | 12 | 39.961 | <0.001 |  | <0.001 |
|  |  | TEST3.SM |  | 14 | 17.333 | 0.239 |  |  |
|  |  | TEST2.CL |  | 11 | 8.158 | 0.699 |  |  |
|  |  | Sum of TESTs | 1.731 | 50 | 86.558 | 0.001 |  |  |
|  |  |  |  |  |  |  |  |  |
|  | c4_3 | TEST3.SR |  | 13 | 13.943 | 0.378 | 0.011 |  |
|  |  | TEST2.CT |  | 12 | 55.805 | <0.001 |  | <0.001 |
|  |  | TEST3.SM |  | 14 | 26.009 | 0.026 |  |  |
|  |  | TEST2.CL |  | 12 | 14.481 | 0.271 |  |  |
|  |  | Sum of TESTs | 2.162 | 51 | 110.238 | <0.001 |  |  |
|  |  |  |  |  |  |  |  |  |
|  | c4_4 | TEST3.SR |  | 13 | 23.962 | 0.032 | <0.001 |  |
|  |  | TEST2.CT |  | 12 | 32.383 | 0.001 |  | <0.001 |
|  |  | TEST3.SM |  | 15 | 22.797 | 0.089 |  |  |
|  |  | TEST2.CL |  | 11 | 18.416 | 0.072 |  |  |
|  |  | Sum of TESTs | 1.913 | 51 | 97.558 | <0.001 |  |  |
|  |  |  |  |  |  |  |  |  |
|  | c4_5 | TEST3.SR |  | 13 | 23.962 | 0.031 | <0.001 |  |
|  |  | TEST2.CT |  | 12 | 32.383 | 0.001 |  | <0.001 |
|  |  | TEST3.SM |  | 15 | 22.797 | 0.089 |  |  |
|  |  | TEST2.CL |  | 11 | 18.416 | 0.072 |  |  |
|  |  | Sum of TESTs | 1.913 | 51 | 97.558 | <0.001 |  |  |
|  |  |  |  |  |  |  |  |  |
|  |  |  |  |  |  |  |  |  |
|  | **Case study** | | | | | | | |
|  | **GOF tests using U-CARE** | | | | | | | |
|  | data set | Tests | c-hat | df | quad chi2 | p-value | P-value for transience | P-value for trap dependence |
|  |  |  |  |  |  |  |  |  |
|  | **Vulture** | TEST3.SR |  | 16 | 24.601 | 0.077 | 0.002 |  |
|  |  | TEST2.CT |  | 53 | 487.068 | <0.001 |  | 0.134 |
|  |  | TEST3.SM |  | 11 | 14.601 | 0.202 |  |  |
|  |  | TEST2.CL |  | 62 | 101.634 | 0.001 |  |  |
|  |  | Sum of TESTs | 4.422 | 142 | 627.903 | <0.001 |  |  |
|  |  |  |  |  |  |  |  |  |
|  | **Penguin** | TEST3.SR |  | 6 | 10.747 | 0.097 | 0.027 |  |
|  |  | TEST2.CT |  | 5 | 37.292 | <0.001 |  | 0.610 |
|  |  | TEST3.SM |  | 8 | 18.906 | 0.015 |  |  |
|  |  | TEST2.CL |  | 9 | 16.979 | 0.049 |  |  |
|  |  | Sum of TESTs | 2.997 | 28 | 83.924 | <0.001 |  |  |
